# Supplementary material for: Rational Construction of C@Sn/NSGr Composites as Enhanced Performance Anodes for Lithium Ion Batteries
Source: Nanomaterials (Basel). 2023 Jan 9;13(2):271. doi: 10.3390/nano13020271 (PMC9861279; doi:10.3390/nano13020271)
Supplement: Supplementary file 1 [file nanomaterials-13-00271-s001.zip › nanomaterials-2063355-supplementary.pdf]

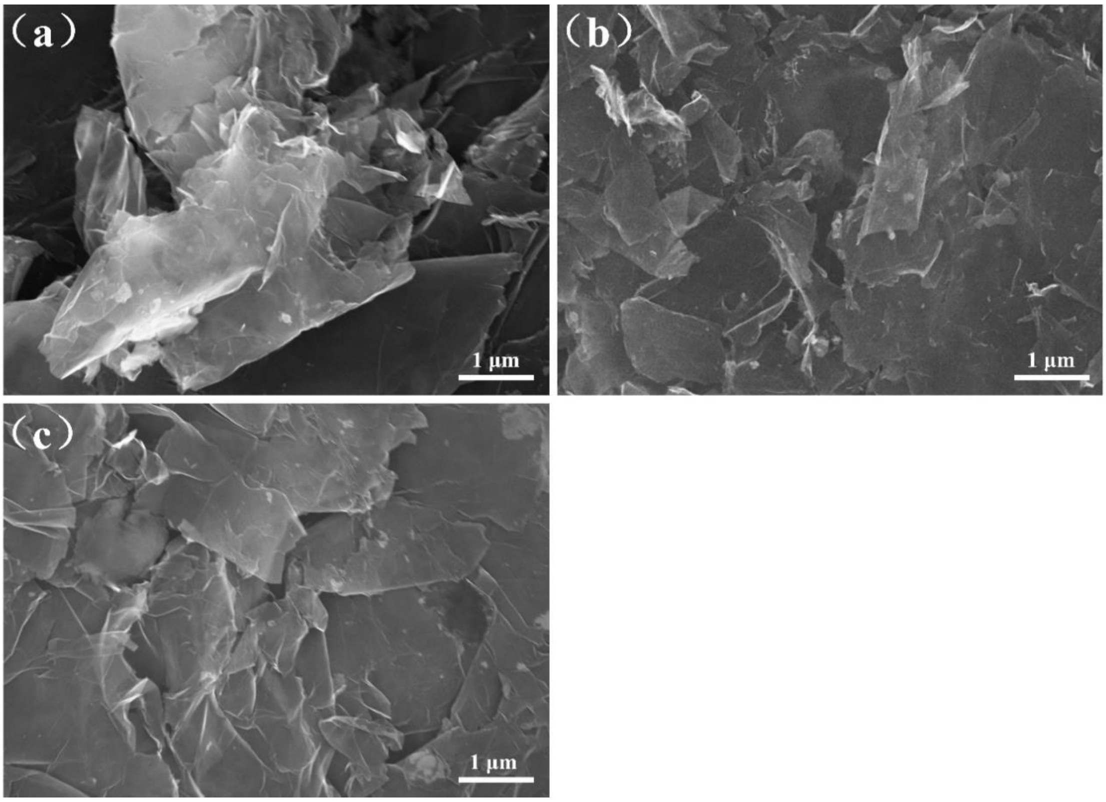


**Figure S1.** SEM images of Sn/NSGr−5 (**a**), Sn/NSGr−7.5 (**b**), and Sn/NSGr−10 (**c**).

**Table S1.** Results of elemental analysis on C@Sn/NSGr−7.5 by EDS.

| Element | Weight Ratio(%) | Atomic Ratio(%) |
|---|---|---|
| C | 88.13 | 94.9 |
| N | 0.42 | 3.01 |
| O | 5.37 | 4.34 |
| S | 0.20 | 0.08 |
| Sn | 6.30 | 0.69 |

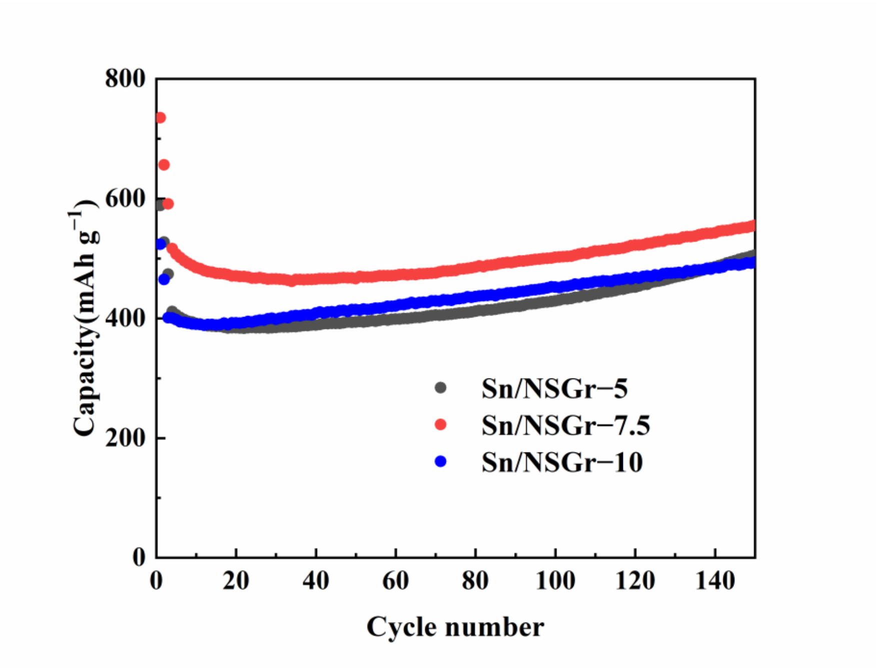


**Figure S2.** Cyclic properties of Sn/NSGr−5, Sn/NSGr−7.5 and Sn/NSGr−10 at 0.1 A g$^{-1}$.

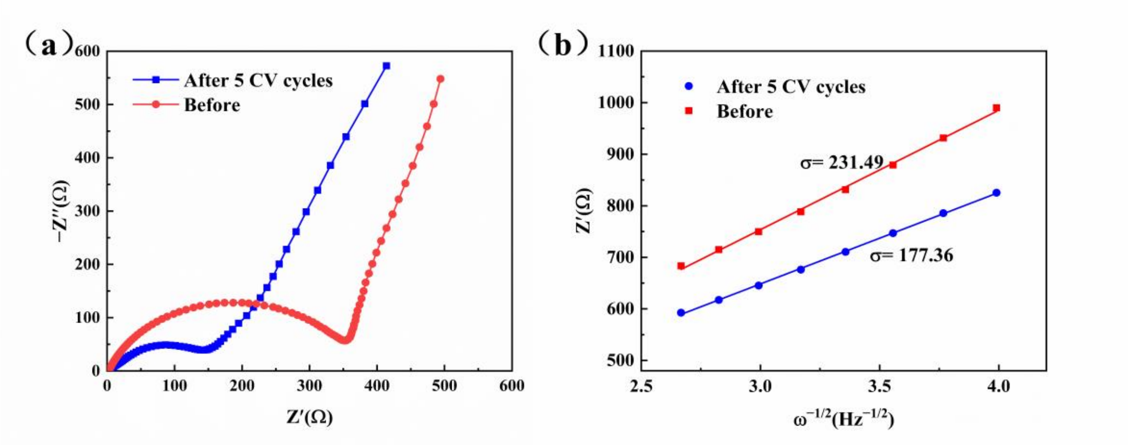


**Figure S3.** Nyquist plots before and after 5 CV cycles of C@Sn/NSGr−7.5 (**a**), the relation of the Z'−ω$^{-1/2}$ curves in the low−frequency region of C@Sn/NSGr−7.5 (**b**).
